# Ethnic variation in the activity of lipid desaturases and their relationships with cardiovascular risk factors in control women and an at-risk group with previous gestational diabetes mellitus: a cross-sectional study

**DOI:** 10.1186/1476-511X-12-25

**Published:** 2013-03-06

**Authors:** Robert G Gray, Eleni Kousta, Mark I McCarthy, Ian F Godsland, Soundarajan Venkatesan, Victor Anyaoku, Desmond G Johnston

**Affiliations:** 1 Diabetes Endocrinology and Metabolic Medicine, Faculty of Medicine, Imperial College London, St. Mary’s Campus, Room G1, Norfolk Place, London W2 1PG, UK; 2 Diabetes and Nutritional Sciences Division, School of Medicine, King’s College London, London, UK; 3 Oxford Centre for Diabetes, Endocrinology and Metabolism, University of Oxford, Oxford, UK

**Keywords:** Ethnicity, Lipids, Blood pressure, Insulin resistance, Stearoyl-CoA desaturase, Delta 6 desaturase, Delta 5 desaturase, Fatty acids, Desaturase activities, Triglycerides, HDL cholesterol, Insulin resistance

## Abstract

**Background:**

Lipid desaturase enzymes mediate the metabolism of fatty acids to long chain polyunsaturated fatty acids and their activities are related to metabolic risk factors for Type 2 diabetes (T2DM) and coronary heart disease (CHD). There are marked ethnic differences in risks of CHD and T2DM but little is known about ethnic differences in desaturase activities.

**Methods:**

Samples from a study of CVD risk in women with previous gestational diabetes were analysed for percentage fatty acids in plasma free fatty acid, triglyceride, cholesterol ester and phospholipid pools for 89 white European, 53 African Caribbean and 56 Asian Indian women. The fatty acid desaturase activities, stearoyl-CoA desaturase (SCD, calculated separately for C16 and C18 fatty acids), delta 6 desaturase (D6D) and delta 5 desaturase (D5D) were estimated from precursor-to-product ratios and their relationships with adiposity, blood pressure, cholesterol, triglycerides, HDL cholesterol and insulin sensitivity explored. Ethnic differences in desaturase activities independent of ethnic variation in risk factor correlates of desaturase activities were then identified.

**Results:**

There was significant ethnic variation in age, BMI, waist circumference, blood pressure, serum triglycerides and HDL cholesterol concentrations and insulin resistance. Desaturase activities showed significant correlations, independent of ethnicity, with BMI, waist circumference, triglycerides and HDL cholesterol. Independent of ethnic variation in BMI, waist circumference, triglycerides and HDL cholesterol, SCD-16 activity, calculated from each of the four lipid pools measured, was 18–35 percent higher in white Europeans than in African Caribbeans or Asian Indians (all p < 0.001). Similar, though less consistent differences were apparent for SCD-18 activity. Also independently of risk factor variation, but specifically when calculated from the cholesterol ester and phospholipid, pools, D6D activity was significantly lower in Asian Indians, and D5D activity higher in African Caribbeans.

**Conclusions:**

Significant ethnic differences exist in desaturase activities, independently of ethnic variation in other risk factors. These characteristics did not accord with higher risk of T2DM among African Caribbeans and Asian Indians nor with lower risk of CHD among African Caribbeans but did accord with the higher risk of CHD in Asian Indians.

## Background

In the U.K., there are marked differences in rates of Type 2 diabetes mellitus (T2DM) and coronary heart disease (CHD) among those of Asian Indian, African Caribbean and white European ethnic origin. Asian Indian populations have a 4-fold greater risk of T2DM and a 1.5 times greater risk of CHD than their white European counterparts [1,2] and among women with T2DM the differential in CHD between South Asians and white Europeans may be even greater [3,4]. Rates of T2DM are 3-fold higher among African Caribbeans [1,5] but, in contrast to Asian Indians, African Caribbeans have a 1.5 times *lower* risk of CHD than white Europeans [3,6,7] and appear to be protected from the expected effects on CHD risk of their higher rates of T2DM and hypertension [5] and insulin resistance [8]. Differences in triglyceride metabolism [9] or body fat distribution [10] may contribute to these differences.

In humans, a key lipid desaturase enzyme is delta-9 desaturase, or stearoyl-CoA desaturase (SCD), which is responsible for conversion of saturated fatty acids to their monounsaturated equivalents. SCD is, therefore, a key enzyme in the control of triglyceride synthesis [11], which requires that at least one site on the glycerol backbone of the triglyceride molecule is occupied by a mono-unsaturated fatty acid [12]. Also in humans, the supply of polyunsaturated fatty acids is dependent on dietary intake or endogenous synthesis from the essential fatty acids, linoleic (18:2 n-6) or alpha linolenic (18:3 n-3) acid. Delta-6 and delta-5 desaturase (D6D and D5D, respectively) activities are responsible for the necessary desaturations. There is evidence that SCD, D6D and D5D activities can be associated with variation in risk factors for T2DM and CVD, in particular those relating to adiposity, triglyceride metabolism and insulin sensitivity [13-15].

To assess ethnic variation in lipid desaturase activities, we have measured the proportions of individual fatty acids in plasma free (non-esterified) fatty acid (FF), triglyceride (TG), cholesterol ester (CE), and phospholipid (PL) pools and estimated desaturase activities according to fatty acid precursor-to-product ratios in each pool. These measurements have been made in control and affected women from Asian Indian, African Caribbean and white European groups, who were participating in a study of T2DM and CHD risk following previous gestational diabetes mellitus (pGDM). We have then explored relationships between desaturase activities and adiposity, body fat distribution, blood pressure, lipids and lipoproteins and insulin sensitivity.

## Results

### Inter-relationships between desaturase activities in different lipid pools

There were significant correlations (R = 0.48–0.68, p < 0.001) between SCD-16 activity in FF, TG, CE and PL compartments whereas SCD-18 activity was only correlated between FF, CE and PL compartments (R = 0.22–0.30, p < 0.01). D6D activity correlated between TG, CE and PL compartments (R = 0.24–0.59, p < 0.001) and D5D activity only between CE and PL compartments (R = 0.39, p < 0.001); n-3 desaturase activity did not correlate between compartments. There were significant positive correlations between SCD-16 and SCD-18 activity in each lipid pool (R = 0.23 to 0.61, p < 0.01). There were significant negative correlations between D6D and D5D activity in each lipid pool (R = −0.26 to −0.36, p < 0.001).

### Significant risk factor variation between the three ethnic groups

Significant variation between the three different ethnic groups was detected in age (decreasing in the order: white European, African Caribbean and Asian Indian), BMI, waist circumference and systolic and diastolic BP (highest in African Caribbeans), serum triglycerides (highest in Asian Indians), HDL cholesterol (lowest in Asian Indians) and HOMA-IS (lowest in African Caribbeans) (Table 1).

**Table 1:** Characteristics by ethnic group

	**White European n = 89**	**African Caribbean n = 53**	**Asian Indian n = 56**	**ANOVA p**
Age	38 (34,41)	36 (32,38) ^*^	34 (31,38) ^§^	0.002
pGDM (%)	42	45	50	0.6
BMI	23.0 (21.2,27.2)	28.8 (25.3,32.6) ^§^	24.9 (22.7,28.4)	<0.001
Waist Circumference	0.76 (0.71,0.86)	0.89 (0.80,0.98) ^†^	0.81 (0.74,0.89)	0.004
Waist Hip Ratio	0.78 (0.74,0.82)	0.82 (0.76,0.86)	0.80 (0.76,0.87)	0.1
Systolic BP (mmHg)	113.7 (14.6)	120.1 (15.5)	110.8 (13.6)	0.009
Diastolic BP (mmHg)	70.2 (9.4)	78.7 (19.0) ^†^	70.5 (9.7)	0.001
Cholesterol (mmol/l)	4.5 (4.1,5.0)	4.5 (4.0,5.2)	5.0 (4.3,5.4)	0.1
Triglycerides (mmol/l)	0.80 (0.65,1.19)	0.79 (0.65,1.01)	1.22 (0.82,1.81) ^§^	<0.001
HDL cholesterol (mmol/l)	1.28 (1.09,1.43)	1.22 (1.04,1.50)	1.01 (0.86,1.24) ^§^	<0.001
Glucose (mmol/l)	4.8 (4.6,5.2)	4.8 (4.6,5.2)	5.0 (4.6,5.4)	0.8
HOMA-IS	78 (41,145)	45 (18,73) *	68 (33,206)	0.02

Medians and interquartile ranges or percentages are shown (or, for blood pressures, mean and standard deviation). Significant differences relative to white Europeans were derived post-hoc for variables showing significant ANOVA between group variation.

Significant differences relative to white Europeans: ^*^ p < 0.05; ^†^ p < 0.01; ^§^ p < 0.001.

### Independent relationships between risk factors and desaturase activities

SCD-16 and D6D activities showed significant, positive relationships, independent of ethnicity, with BMI, waist circumference and triglycerides, primarily in activities calculated from the CE and PL pools; D6D activity also showed significant negative associations with HDL cholesterol, also in the CE and PL pools (Table 2). D5D activity showed significant, negative relationships, independent of ethnicity, with BMI, waist circumference and triglycerides, and positive relationships with HDL cholesterol, primarily in activity calculated from the PL pools. Net n-3 desaturase activity, calculated from the CE and PL pools was positively associated with HDL cholesterol levels.

**Table 2:** Independent associations between risk factors and desaturase activities in 5 lipid pools in regression models that also included African Caribbean ethnicity and Asian Indian ethnicity (partial correlation coefficients)

	**BMI**	**Waist**	**Waist hip ratio**	**Systolic BP**	**Diastolic BP**	**Chol**	**Trig**	**HDL chol**	**Glucose**	**HOMA-IS**
**Stearoyl-CoA desaturase C16**										
FF	0.20^†^	0.16*	0.00	0.09	0.04	−0.07	0.06	0.00	0.04	0.00
TG	0.09	0.05	−0.01	0.07	0.04	−0.06	0.09	−0.01	−0.01	0.07
CE	0.29 ^§^	0.26^§^	0.22^†^	0.07	0.01	0.14	0.39^§^	−0.10	0.10	−0.03
PL	0.27 ^§^	0.18*	−0.02	0.12	0.06	0.03	0.20^†^	0.01	0.12	−0.09
**Stearoyl-CoA desaturase C18**										
FF	0.21^†^	0.18*	0.06	0.11	0.14	−0.13	0.02	−0.02	0.09	−0.03
TG	−0.11	−0.15*	−0.12	−0.01	0.01	−0.08	−0.03	−0.05	−0.03	0.11
CE	−0.07	−0.07	0.09	0.00	−0.05	0.05	0.01	0.01	−0.03	0.14
PL	0.01	−0.05	−0.08	0.09	0.01	−0.13	−0.17*	0.13	−0.04	0.03
**n-6 Δ-6 desaturase**										
FF	0.01	0.07	0.04	0.05	0.08	−0.05	0.05	−0.09	−0.02	−0.08
TG	0.10	0.12	0.12	−0.01	−0.04	0.16*	0.10	0.11	0.04	−0.13
CE	0.26^§^	0.23^†^	0.12	−0.02	0.01	0.03	0.28^§^	−0.19^†^	−0.04	−0.12
PL	0.20^†^	0.18*	0.14	0.06	−0.10	0.11	0.32^§^	−0.17*	−0.06	0.11
**n-6 Δ-5 desaturase**										
FF	−0.02	−0.01	−0.01	0.00	0.07	0.14	0.12	0.14	0.01	0.03
TG	0.03	−0.02	−0.08	0.11	0.16*	−0.17*	−0.35^§^	0.13	−0.04	0.25^§^
CE	−0.12	−0.13	−0.15*	0.15	0.12	0.02	−0.11	0.15*	0.20^†^	0.04
PL	−0.22^†^	−0.21^†^	−0.21^†^	−0.01	−0.06	−0.16*	−0.37^§^	0.24^†^	0.06	0.01
**n-3 Δ desaturase**										
FF	0.00	0.04	−0.01	−0.07	−0.05	0.06	0.14	−0.02	−0.10	−0.08
TG	0.14	0.12	0.07	−0.04	0.01	−0.03	−0.08	0.02	−0.08	0.12
CE	0.05	−0.01	0.13	−0.08	−0.11	0.04	−0.14	0.22^†^	−0.04	−0.08
PL	−0.08	−0.02	0.09	0.02	−0.11	0.09	−0.04	0.16*	−0.03	−0.12
African Caribbean	0.24^†^–0.34 ^§^	0.18*–0.32 ^§^	0.09–0.22^†^	0.15–0.20*	0.22^†^–0.27^†^	−0.07–0.04	−0.13–0.11	−0.02–0.08	−0.02–0.07	−0.22^†^–−0.13
Asian Indian	0.06–0.14*	0.01–0.08	−0.05–0.20^†^	−0.10–−0.01	−0.02–0.03	0.04–0.15*	0.20^†^–0.40^§^	−0.34^§^–−0.21^†^	−0.02–0.02	−0.03–0.07

Abbreviations: *TF* total fatty acids; *FF* free fatty acids; *TG* triglycerides; *CE* cholesterol esters; *PL* phospholipids. Significances: * p < 0.05 (borderline); ^†^ p < 0.01; ^§^ p < 0.001.

African Caribbean ethnicity was associated with younger age and independently associated with increased BMI, waist hip ratio and diastolic BP and decreased waist circumference, serum triglycerides, plasma glucose and HOMA-IS. Asian Indian ethnicity was associated with younger age and independently associated with increased BMI, waist hip ratio and serum triglycerides and decreased waist circumference (as shown in Table 2, waist circumference was relatively high in African Caribbean and Asian Indian groups-further regression analyses (results not shown) established the negative associations between ethnicity and waist circumference in partial correlation reflected the consequences of including BMI and waist hip ratio as partial correlates, which, together, would have largely accounted for variation in total and central adiposity, thus leaving a narrow waist as an independent characteristic of these two ethnic groups). Increasing age tended to be associated with increased waist hip ratio and pGDM with increased triglyceride and glucose levels. Neither age nor pGDM showed any consistent relationships with desaturase activities (results not shown).

### Independent effects of ethnicity on desaturase activities

The correlates of variation in desaturase activities, independent of ethnicity (identified in Table 2) were: BMI, waist circumference and serum triglyceride and HDL cholesterol levels. Since the three ethnic groups varied significantly in the these four variables, the magnitude of any independent effect of ethnicity on desaturase activities was quantified relative to desaturase activities in white Europeans (the largest group recruited) by multiple linear regression analysis, with each desaturase activity predicted by African Caribbean or Asian Indian ethnicity plus BMI, waist circumference and serum triglyceride and HDL cholesterol levels (Table 3).

**Table 3:** Product: precursor ratio desaturase activities in 5 lipid pools with effects of African Caribbean and Asian Indian ethnicity relative to White European derived from linear regression models incorporating ethnicity and potential confounding variables

	**White European n = 89 (median, IQ range)**	**African-Caribbean n = 53 (coefficient, 95%CI)**	**Asian Indian n = 56 (coefficient, 95%CI)**
**Stearoyl-CoA desaturase C16**			
FF	0.15 (0.12,0.20)	−0.05 (−0.07,−0.04) ^§^	−0.04 (−0.05,−0.02) ^§^
TG	0.17 (0.14,0.20)	−0.04 (−0.05,−0.03) ^§^	−0.03 (−0.04,−0.02) ^§^
CE	0.25 (0.20,0.30)	−0.08 (−0.10,−0.04) ^§^	−0.06 (−0.09,−0.03) ^§^
PL	0.02 (0.02,0.03)	−0.007 (−0.010,−0.004) ^§^	−0.005 (−0.008,−0.002) ^§^
**Stearoyl-CoA desaturase C18**			
FF	3.8 (3.0,5.0)	−0.14 (−0.24,0.03) *	−0.06 (−0.17,0.05)
TG	11.3 (9.7,13.8)	−0.23 (−0.36,−0.09) ^†^	−0.15 (−0.30,−0.01) *
CE	20.3 (16.8,23.5)	−0.13 (−0.24,−0.02) *	−0.09 (−0.21,,0.03)
PL	0.84 (0.73,0.94)	−0.13 (−0.17,−0.09) ^§^	−0.13 (−0.16,−0.09) ^§^
**n-6 Δ-6 desaturase**			
FF	0.00 (0.00,0.01)	0.003 (−0.002,0.008)	−0.002 (−0.007,0.004)
TG	0.01 (0.01,0.02)	0.001 (−0.002,0.005)	0.002 (−0.002,0.006)
CE	0.01 (0.01.0.01)	−0.001 (−0.002,0.001)	−0.003 (−0.004,−0.001) ^†^
PL	0.09 (0.07,0.11)	−0.010 (−0.021,−0.001) *	−0.025 (−0.036,−0.014) ^§^
**n-6 Δ-5 desaturase**			
FF	2.1 (0.0,12.5)	0.16 (−0.31,0.62)	0.51 (0.00,1.01) *
TG	4.4 (3.2,5.5)	0.17 (−0.01,0.35)	0.01 (−0.19,0.20)
CE	8.9 (7.4,10.8)	0.29 (0.13,0.45) ^†^	0.14 (−0.04,0.32)
PL	2.9 (2.2,3.5)	0.13 (0.04,0.22) ^†^	0.12 (0.02,0.22) *
**n-3 Δ desaturase**			
FF	0.00 (0.00,0.20)	0.22 (−0.00,0.44)	−0.10 (−0.34,0.10)
TG	0.18 (0.11,0.30)	0.16 (0.03,0.29) *	0.09 (−0.05,0.23)
CE	1.28 (0.92,1.79)	0.12 (−0.30,0.55)	0.28 (−0.18, 0.75)
PL	2.78 (1.79,4.43)	−0.03 (−0.33,0.26)	−0.09 (−0.40,0.23)

Abbreviations: *TF* total fatty acids; *FF* free fatty acids; *TG* triglycerides; *CE* cholesterol esters; *PL* phospholipids.

Significant, independent effects of ethnicity on desaturase activity were identified with regression models incorporating ethnicity and potential confounding variables, according to the general structure: *desaturase activity = b1.(African Caribbean ethnicity) + b2.(Asian Indian ethnicity) + b3.(BMI) + b4.(waist circumference) + b5.(triglycerides) + b6.(HDL cholesterol).*

Significances * p < 0.05; ^†^ p < 0.01; ^§^ p < 0.001 refer to the significance of coefficients b1 and b2 and give the significance of the effects of African Caribbean and Asian Indian ethnicity, respectively, relative to white European ethnicity.

With activity calculated on the basis of each lipid pool evaluated, African Caribbean and Asian Indian ethnicities were independently associated, consistently and highly significantly, with lower SCD-16 activity. Depending on the lipid pool used in the calculation, SCD-16 activity was 24–35 percent lower (p < 0.001) in African Caribbeans and 18–27 percent lower (p < 0.001) in Asian Indians compared with white Europeans. Although less consistent between lipid pools, the same pattern was seen with SCD-18 activity. Asian Indian ethnicity was associated with lower D6D activity, with activity calculated from the CE and PL pools and African Caribbean ethnicity was associated with higher D5D activity, also with activity calculated from the CE and PL pools.

## Discussion

Lipid desaturase enzymes have a wide tissue distribution and we evaluated desaturase activities on the basis of fatty acid compositions in a range of plasma lipid pools. Fatty acids in the FF pool would be expected to reflect adipose tissue metabolism, being largely the product of adipose tissue lipolysis and there is previous evidence for this with respect to SCD-16, SCD-18 and D5D activities [15]. In the TG pool, fatty acids would be expected to reflect fatty acid metabolism in the liver (following VLDL secretion) and be influenced by both recent meal content and by endogenous metabolism. Fatty acids in the CE pool would also be expected to reflect hepatic metabolism, but would relate to diet over preceding weeks to months. The fatty acid composition of the PL pool is more difficult to interpret [15] and it is, therefore, of interest that there were strong correlations between desaturase activities derived from CE and PL pool fatty acid compositions. Moreover, in general, ethnic differences in desaturase activities and associations between desaturase activities and metabolic risk factors were most apparent when desaturase activities were calculated from the fatty acid compositions of the CE and PL pools.

Nevertheless, with standardization for potential confounders, lower SCD-16 activity was apparent in *all* pools in African Caribbean and Asian Indian women relative to white Europeans. Similar, though slightly less consistent differences were seen with SCD-18 activity. Overall, these observations strongly support the conclusion that white European women have higher SCD activity than Asian Indian or African Caribbean women. Genetic variation has been reported in *Scd-1*[16] but differences in polymorphism frequencies between Asian Indians, African-Caribbeans and white Europeans do not appear to have been described.

We found D6D activity to be significantly lower in Asian Indian women relative to white Europeans. D6D metabolises the first step in conversion of the essential fatty acids linoleic (18:2 n-6) and alpha linolenic (18:3 n-3) to longer chain, polyunsaturated fatty acids and our observations are consistent with the proposal that reduced D5D activity might be responsible for lower polyunsaturated fatty acid concentrations in Asian Indians [17]. Genetic variation might underlie the lower D6D activity in Asian Indians but definitive studies of ethnic variation in the *Fads2* gene that codes for D6D have yet to be carried out. D5D activity was significantly higher in African Caribbean women relative to white Europeans. D5D metabolises eicosatrienoic acid to arachidonic acid and higher arachidonic acid levels have been reported previously in people of Black African origin [18]. D5D is coded by the *Fads1* gene and it has been suggested that the higher arachidonic acid levels in African Caribbeans could reflect ethnic differences in *Fads1* genotype [19,20]. However, as with the *Fads2* gene, ethnic variation in *Fads1* genotype has yet to be explored in depth.

Recent evidence has confirmed an association between high levels of C16 and C18 saturated fatty acids in the plasma PL pool and increased risk of CHD and between high levels of n-6 polyunsaturated fatty acids and decreased risk of CHD [21]. The relatively low SCD activity in Asian Indian women might be expected to be associated with higher C16 and C18 fatty acid levels and the relatively lower D6D activity with lower n-6 polyunsaturated fatty acid levels. Both characteristics we identified in Asian Indians would, therefore, accord with their elevated risk of CHD. However, no such consistencies could be inferred with regard to the relatively low risk of CHD in African Caribbean women.

Independently of ethnicity, SCD-16 activity was positively associated with BMI, waist circumference and serum triglycerides. Positive associations between SCD activity and adiposity have been reported previously [13,14], and a positive association with serum triglyceride concentrations is well-documented [11,22]. We found no evidence for an association between SCD activity and insulin sensitivity but previous studies have reported positive [13] or negative [15] associations between SCD activity and insulin sensitivity so this issue remains unresolved. The independent associations we observed between D6D and D5D activity and risk factors were generally similar to those described by others [13,15], with positive associations between D6D activity, adiposity and triglycerides and negative associations between these risk factors and D5D activity.

Previous studies have found a positive association between insulin sensitivity and D5D activity, calculated from skeletal muscle phospholipid composition [23,24] but we found little evidence for associations between desaturase activities calculated from plasma fatty acids and either glucose levels or insulin sensitivity. The single positive association noted between insulin sensitivity and D5D activity derived from fatty acid ratios in the TG pool and may have been a chance finding. With regard to ethnic variation in risk of T2DM, it could be speculated that the lower SCD activities in African Caribbeans and Asian Indians might contribute to increased levels of saturated fatty acids, increased levels of which may be associated with increased risk of T2DM [25,26]. However, given the overall lack of association between diabetes risk factors and desaturase activities, it seems unlikely that ethnic variation in desaturase activity contributes to ethnic variation in risk of T2DM.

Our study has limitations in that it was restricted to a subgroup of a larger cohort [27] and both the African Caribbean and Asian Indian women were slightly but significantly younger than the Europeans. Previous studies [1,28-30], including the full cohort study from which the present analysis was drawn [27], report that, compared to European women, African Caribbean women have higher BMI, higher or similar systolic and diastolic BP, similar HDL cholesterol, lower triglycerides and decreased insulin sensitivity, whereas Asian Indian women have higher or similar BMI, similar blood pressure, lower or similar HDL cholesterol, higher or similar triglycerides and lower or similar insulin sensitivity. Ethnic differences in these measures in the present study were, therefore, broadly similar to previous findings, although in our smaller subgroup we found no significant difference in triglyceride levels between European and African Caribbean women. The systematic multivariable analysis we adopted was designed to minimize any effects of these differences in risk factors on relationships between desaturase activities and ethnicity. A further potential limitation was that the study from which the present analysis derives did not include recording of dietary information. There is undoubtedly considerable ethnic variation in dietary habits, but it is important to note that our analysis concerned measures derived from precursor-to-product ratios, which should be relatively uninfluenced by variation in absolute levels of either precursors or products.

A strength of our study was that desaturase activities were calculated on the basis of fatty acid compositions in four different lipid pools. It was, therefore, possible to evaluate consistencies between pools in the calculated desaturase activities and we were able to demonstrate complete consistency for elevated SCD-16 activity in white Europeans compared with African Caribbeans or Asian Indians. These differences have not been investigated previously, but their strength and consistency in our analysis justifies further, dedicated confirmatory studies. By contrast, reduced D6D activity in Asian Indians and elevated D5D activity in African Caribbeans was restricted to activities calculated from the CE and PL pools. This suggests ethnic variation in desaturase activities in specific tissues, most likely the liver, and justifies further investigations of ethnic variation in tissue-specific desaturase activities.

## Conclusions

Despite low SCD activities in African Caribbeans and Asian Indians possibly contributing to a tendency to higher C16 and C18 saturated fatty acid levels, it seems unlikely, given the absence of any association between desaturase activities and plasma glucose concentrations or insulin resistance, that ethnic variation in desaturase activities contributes to ethnic variation in risk of T2DM. Similarly, variation in desaturase activities showed no consistency with the lower risk of CHD in African Caribbeans. However, the relatively low D5D activity we observed in Asian Indian women suggests a contribution from lower desaturase activities to the higher risk of CHD in Asian Indians.

## Methods

### Study design

Samples for estimation of desaturase activities were from a previously-described study of insulin sensitivity and secretion in women with pGDM [27,31]. In brief, women with pGDM were identified from West London databases with recruitment at 20.0 months (geometric mean, 95% CI 18.2–22.1 months) following delivery. Entry was based on WHO criteria for glucose intolerance during pregnancy and on the women being of European, Asian-Indian (India, Pakistan, Bangladesh or Sri Lanka), or African-Caribbean ethnicity. Entry criteria for a control group were: normal glucose levels throughout the index (and other) pregnancies, normal fasting glucose and HbA_1_c at recruitment and European, Asian-Indian or African-Caribbean ethnicity. Controls were recruited at a mean of 21.6 (20.0–23.4) months after the index pregnancy. Ethical approval was from the UK Multicentre Research Ethics Committee and local ethical committees and informed consent was obtained. Measurements included anthropometry (height, weight, waist and hip circumferences), resting blood pressure, fasting serum cholesterol, triglycerides and HDL cholesterol, and plasma glucose and insulin. The original study included 368 women with pGDM and 482 controls. For fatty acid analysis, samples from 198 women were selected from both groups at random.

### Determination of lipid desaturase activities

For determination of plasma lipid fatty acid compositions, blood was collected into an EDTA tube, mixed thoroughly and then centrifuged at 2500 g , 4°C for 15 min. An aliquot of the supernatant plasma was withdrawn and the lipids extracted in accordance with the Folch method. Free fatty acid (FF), triglyceride (TG), cholesterol ester (CE) and phospholipid (PL) lipid species were isolated by thin layer chromatography and the lipid fatty acids trans-methylated and their compositions in each lipid species pool measured by gas chromatography, as previously described [32]. The composition of each fatty acid in each pool was expressed as a percentage of the total fatty acids in that pool.

Lipid desaturase activities were derived as product-to-precursor ratios for the fatty acids metabolised and produced by each desaturase. Accordingly, the activity of the delta-9 desaturase, SCD, was derived from the palmitic acid-to-palmitoleic acid ratio (16:1/16:0: SCD-16) or from the stearic acid-to-oleic acid ratio (18:1/18:0: SCD-18). SCD activities were calculated for each lipid pool. Similarly, D6D activity was derived from the gamma linolenic-to-linoleic acid ratio (18:3n-6/18:2n-6) in each pool and D5D activity from the arachidonic-to-eicosatrienoic acid ratio (20:4n-6/20:3n-6) in each pool. We also estimated the net n-3 delta desaturase activity from the eicosapentaenoic-to-eicosahexaenoic acid ratio (20:5n-3/18:3n-3). Some individual n-3 polyunsaturated fatty acids were undetectable. In these cases, a value 50 percent of the minimum value encountered was substituted for calculation of desaturase activities.

### Data analysis

Insulin sensitivity, HOMA-IS, was calculated from the homeostasis model assessment formula: HOMA-IS = 1 / ([plasma glucose (mmol/l) × plasma insulin (mU/l) / 22.5) [33]. Summary measures were derived as medians and inter-quartile ranges. For statistical testing, variables were log-transformed as necessary to normalize their distributions and parametric statistical tests were used throughout. Where log-transformation was applied, a value of 1 was added to all values for those variables in which values of zero were encountered. Dummy variables were assigned for pGDM status and African Caribbean and Asian Indian ethnicity. Four analysis were undertaken:

1) Inter-relationships between desaturase activities calculated from lipid proportions in different lipid pools and inter-relationships between different desaturase activities were explored by Pearson correlation.

2) Significant variation between the three ethnic groups in the participant characteristics and the risk factors: age, pGDM, BMI, waist circumference, waist hip ratio, systolic and diastolic BP, serum cholesterol, triglycerides and HDL cholesterol, fasting plasma glucose and HOMA-IS was detected by ANOVA.

3) Independent relationships between risk factors and desaturase activities were explored by partial correlation.

4) The magnitude of any independent effect of ethnicity on desaturase activities was quantified relative to desaturase activities in white Europeans (the largest group recruited) by multiple linear regression analysis, with each desaturase activity predicted by ethnicity plus those risk factors that were found to be independently associated with desaturase activities in 3) and differed between different ethnic groups.

Combining measurements made in all five pools, the number of different activities measured was 20. Consequently, there were 20 statistical tests for each hypothesis and one significant association would be expected by chance. No correction was made for the number of hypotheses tested, since these were evidence-weighted [34] i.e. previous studies, both mechanistic and observational, had already established the possibility of effects of or on desaturase activities in relation to the variables examined.

## Abbreviations

BMI: Body mass index; CE: Cholesterol ester; CHD: Coronary heart disease; D5D: Delta-5 desaturase; D6D: Delta-6 desaturase; FF: Free (non-esterified) fatty acid; HDL: High density lipoprotein; HOMA-IS: Homeostasis model assessment insulin sensitivity; pGDM: Previous gestational diabetes mellitus; PL: Phospholipid; SCD: Stearoyl-CoA desaturase; T2DM: Type 2 diabetes mellitus; TG: Triglyceride.

## Competing interests

There were no competing interests.

## Authors’ contributions

RGG: study conception and design, acquisition of data, critical review of manuscript. EK: study design, acquisition of data, critical review of manuscript. MIM: study design, acquisition of data, critical review of manuscript. IFG: data analysis, manuscript writing. SV: study conception and design, acquisition of data and critical review of manuscript. VA: acquisition of data and critical review of manuscript. DGJ: study conception and design, manuscript writing. All authors read and approved the final manuscript.
